# Efficacy and limitations of amniotic membrane transplantation in cases of severe ocular surface disorders: retrospective analysis over a 21-year period

**DOI:** 10.1007/s10384-025-01209-z

**Published:** 2025-06-06

**Authors:** Kohei Harada, Hideki Fukuoka, Koji Kitazawa, Yulia Aziza, Tsutomu Inatomi, Tomoyuki Hino, Go Horiguchi, Satoshi Teramukai, Shigeru Kinoshita, Masafumi Uematsu, Takashi Kitaoka, Chie Sotozono

**Affiliations:** 1 Department of Ophthalmology, Kyoto Prefectural University of Medicine, 465 Kajii-cho, Hirokoji-agaru, Kawaramachi-dori, Kamigyo-ku, Kyoto, 602-0841 Japan; 2 Department of Ophthalmology and Visual Sciences, Graduate School of Biomedical Sciences, Nagasaki University, Nagasaki, Japan; 3 Department of Ophthalmology, National Center for Geriatric and Gerontology, Obu, Japan; 4 Department of Biostatistics, Graduate School of Medical Science, Kyoto Prefectural University of Medicine, Kyoto, Japan; 5 Department of Frontier Medical Science and Technology for Ophthalmology, Prefectural University of Medicine, Kyoto, Japan

**Keywords:** Amniotic membrane transplantation (AMT), Severe ocular surface disorders (OSDs), Ocular surface grading score (OSGS), Reoperation, Conjunctival hyperemia

## Abstract

**Purpose:**

To examine the reoperation rate of amniotic membrane transplantation (AMT) and clarify the risk factors for AMT reoperation in severe ocular surface (OS) disorders (OSD).

**Study design:**

Retrospective cohort study.

**Participants:**

We reviewed the medical records of all AMT cases between April 1998 and June 2019 at the Department of Ophthalmology, Kyoto Prefectural University of Medicine, Kyoto, Japan.

**Methods:**

Primary diseases and AMT reoperation rates were examined. In severe OSD cases (Stevens–Johnson syndrome, ocular cicatricial pemphigoid, and chemical/thermal burns), preoperative OS Grading Score (OSGS), surgical procedures combined with AMT, and risk factors for reoperation were investigated and assessed.

**Results:**

Over a period of 21 years and 3 months, 750 AMTs were performed on 664 eyes of 594 cases. AMT was repeated on 51 of those 664 eyes (7.7%), and most frequently performed on 25 out of 196 eyes (12.8%) afflicted with severe OSDs. In severe OSDs, OSGS was significantly higher in the reoperation group compared to no-reoperation group (*P*<0.05), suggesting corneal epithelial defects, conjunctival hyperemia, trichiasis, mucocutaneous junction involvement, and corneal opacity as being risk factors for re-AMT (univariate analysis). In logistic regression analysis, only conjunctival hyperemia was a risk factor, with odds ratios (OR) of 2.65 (95%CI: 1.34–5.22, *P*=0.005). AMT combined with cultivated or donor corneal epithelial transplantation reduced reoperation risk with an OR of 0.92 and 0.63, respectively.

**Conclusions:**

In severe OSD cases, the effect of AMT is limited. Higher OSGSs, especially in conjunctival hyperemia, are associated with a high risk of repeat AMT.

## Introduction

Severe ocular surface (OS) disorders (OSDs) resulting from Stevens-Johnson syndrome (SJS), ocular cicatricial pemphigoid (OCP), and chemical and thermal burns can cause the loss of limbal stem cells. In such cases, conjunctival invasion onto the cornea, persistent epithelial defects, fornix shortening/symblepharon, and corneal opacity often develop, resulting in serious loss of vision [1–4]. Surgical interventions for OSDs such as penetrating keratoplasty (PKP) and lamellar keratoplasty (LKP) do not provide limbal stem cells, resulting in poor postoperative prognosis and a possible complete graft failure following limbal epithelial transplantation (LT)/keratoepithelioplasty (KEP) surgery, even in cases where the donor tissue includes limbal epithelial stem cells.

Amniotic membrane (AM) transplantation (AMT) has been used worldwide since the late 1990s for the treatment of severe OSDs. AM serves as a substrate on which epithelial cells can easily proliferate [5], as the collagen of the basement membrane of AM is very similar to the basement membrane of the conjunctiva and cornea. AM not only acts as a basement membrane to promote epithelialization via the migration of conjunctival cells [6], but also inhibits fibroblast proliferation by suppressing transforming growth factor beta receptors and signaling in the conjunctiva, thus inhibiting inflammation by suppressing neutrophil penetration and proteolytic enzyme activity, which suppresses neovascularization [7–10]. Moreover, one of the most important characteristics of human AM is the lack of the expression of HLA-A, B, and DR, major histocompatibility complex class I (HLA-A and HLA-B) and class II (HLA-DR) antigens [11, 12]. Hence, immune rejection does not occur post AMT and immunosuppression is not necessary [5].

AMT can be combined with corneal epithelial transplantation procedures such as LT and KEP to treat severe OSDs [13, 14]. In such combined surgery, AM acts as a substrate for expanding transplanted corneal limbal epithelial stem cells (LECs) [15]. In conventional AMT combination surgery (AMT combined with LT/KEP, and others), it takes several weeks for the corneal epithelial cells to cover the entire transplanted donor corneal graft. We previously reported the efficacy of cultivated corneal limbal epithelial transplantation (CLET) and cultivated oral mucosal epithelial transplantation (COMET), which combine epithelial-cell transplantation and AM transplantation via the culturing of corneal epithelial cells or oral mucosal epithelial cells on AM to create a multilayered epithelial cell sheet [16, 17]. Both procedures make it possible for the entire cornea to be immediately covered with epithelial cells during surgery, and also provide the anti-inflammatory effect of the AM [17]. In severe OSD cases, total reconstruction of the OS, i.e., both the cornea and conjunctiva, is necessary. AMT can be combined with nearly all limbal transplantation procedures, such as LT, KEP, CLET, and COMET.

Although significant research has been conducted on the efficacy and limitations of AMT, further studies are needed to more fully elucidate its role and limitations in the management of severe OSDs. In this study, we retrospectively examined a large number of severe OSD cases seen over a 21-year period to evaluate the risk factors for reoperation in cases that underwent AMT for substrate purposes.

### Subjects and methods

This study was approved by the Ethics Committee and Institutional Review Board of the Kyoto Prefectural University of Medicine, Kyoto, Japan, and was carried out in accordance with the tenets set forth in the Declaration of Helsinki.

### Study participants

This retrospective cohort study included all cases that underwent AMT between April 1998 and June 2019 at the Department of Ophthalmology, Kyoto Prefectural University of Medicine. We collected and reviewed the medical records of all cases in which AMT was performed, and categorized those cases depending on the primary disease. In addition, the rate of AMT reoperation was compared by disease. Cases in which AMT was performed for the purpose of being a substrate were included, and the cases in which the initial purpose for performing AMT was other than as a substrate (i.e., a patch, a fill-in, etc.) were excluded.

### OS grading score (OSGS) in severe OSD cases

Our previously reported OSGS was used to evaluate the severity of the OSDs in the cornea, conjunctiva, and eyelid regions [18]. Also, the findings of upper and lower fornix shortening were included when assessing the level of conjunctival scarring [19]. In this study, the preoperative OSGS was evaluated for severe OSDs including SJS, OCP, and chemical and thermal burns.

In all cases, the following 12 factors were examined: (1) corneal epithelial defect, (2) palisades of Vogt (POV), (3) conjunctivalization, (4) neovascularization, (5) opacification, (6) keratinization, (7) hyperemia, (8) symblepharon formation, (9) shortening of the upper conjunctival sac, (10) shortening of the lower conjunctival sac, (11) trichiasis, and (12) mucocutaneous junction involvement. Those 12 factors were then evaluated using our OSGS system that divides each component into four levels, i.e., from 0 to 3, according to the severity of the lesion [18, 19].

The OSGS findings were combined to produce a total OSGS ranging from 0 (minimum) to 36 (maximum), with a total OSGS of 36 representing the eyes that were most severely affected. In addition, the correlation coefficients in each component of the OSGS with significant differences were examined.

### Combined surgery on the OS

We examined the specific epithelial transplantation surgical procedures used in conjunction with the initial AMT for the treatment of the severe OSDs. The surgical methods were categorized as COMET, CLET, KEP/LT, and LKP. In these four subgroups, the preoperative OSGS was evaluated; i.e., combined-surgery (+) or (−), and with or without reoperation for AMT.

### Statistical analysis

Continuous variables are expressed as mean ± standard deviation (SD), and categorical variables are expressed as number of cases (%). Comparisons between the no-reoperation group and the reoperation group were performed using the Wilcoxon rank-sum test for continuous variables including the OSGS before the first surgery, and Fisher's exact test for categorical variables.

In addition, we investigated whether or not there was a correlation between each OSGS component. The correlation coefficient was calculated using the Spearman’s rank correlation coefficient. Multivariable logistic regression analysis was used to investigate prognostic factors associated with reoperation and to estimate the odds ratios (OR) and their 95% confidence intervals (CI) for each of factors.

All statistical analysis was conducted using JMP version 14 (SAS Institute, Inc.) and SAS version 9.4 (SAS Institute Inc.). A *P*-value of < 0.05 was considered statistically significant (indicated in bold in Tables 1 and 2).

## Results

### Diseases and reoperation rate

**Fig. 1:**
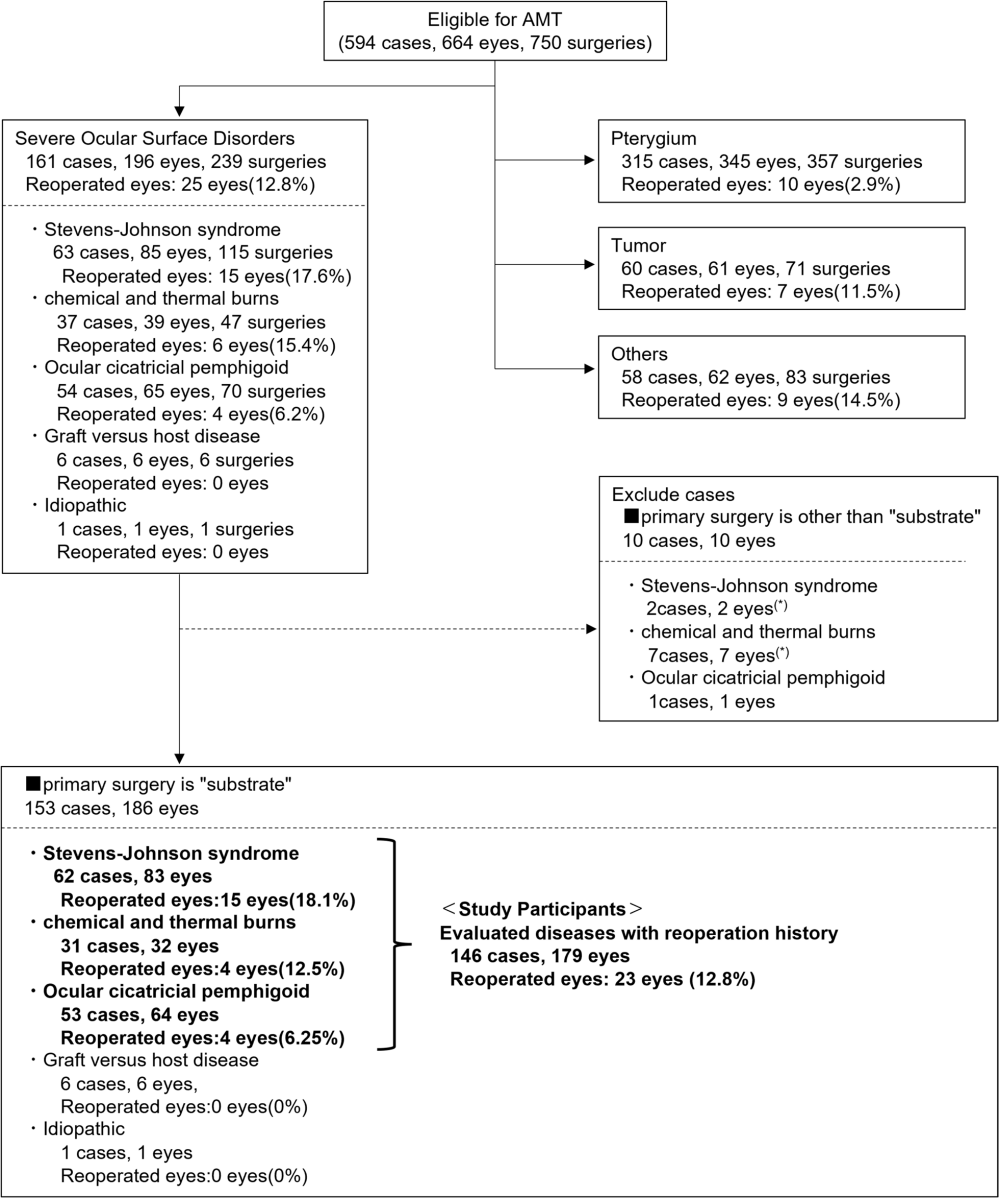
Diagram showing the flow of the study. Over 21 years and 3 months, 750 AMTs were performed on 664 eyes of 594 cases. Of the 664 eyes, 196 with severe OSD underwent AMT. In this study, we targeted severe OSD patients who underwent reoperation with AMT used as a "substrate." *In one case, one eye is included in this study. AMT: amniotic membrane transplantation, OSD: ocular surface disorder, OSGS: ocular surface grading score.

Over a period of 21 years and 3 months, 750 AMTs were performed on 664 eyes of 594 patients [20]. Of the total 750 AMT procedures performed, pterygium was the most common, with 357 pterygium surgical procedures performed on 345 eyes. Moreover, 239 AMT procedures were performed on 196 eyes with a severe OSD, and of those 196 eyes, repeat AMT surgery was performed on 25 (12.8%), a much higher percentage compared to the 10 of the 357 eyes (2.9%) in the pterygium group (Fig. 1).

Among 196 eyes with an OSD, 10 were excluded from the OGSG study in which AM was used as a stuff or a patch. Six eyes with graft-versus-host disease and 1 eye with an idiopathic OSD did not undergo reoperation. Therefore, the OSGS before the initial surgery was evaluated in 179 eyes with an OSD; i.e., 83 eyes with SJS, 64 eyes with OCP, and 32 eyes with chemical and thermal burns (Fig. 1). Of those 179 eyes, a repeat AMT surgery was performed on 23 (12.8%); i.e., 15 (18.1%) of the 83 SJS eyes, 4 (12.5%) of 32 chemical and thermal burn eyes, and 4 (6.25%) of 64 OCP eyes.

Reoperation was performed at the surgeon's discretion in accordance with any worsening symptoms, such as recurrence of symblepharon formation or shortening of the conjunctival sac. In cases with mild symptoms, or in elderly patients who were unable to, or opted not to, undergo surgery, no reoperation was performed.

### Comparison of OSGS between the severe OSD reoperation and non-reoperation groups

Of the 179 eyes with a severe OSD, 23 underwent reoperation (reoperation group) and 156 did not undergo reoperation (no-reoperation group). The mean patient age in the reoperation group and no-reoperation group was of 54.8 ± 14.2 years and 58.2 ± 21.1 years, respectively (Table 1). The mean total OSGS before AMT in the reoperation group and no-reoperation group was 21.5 ± 5.52 and 17.65 ± 5.67, respectively, thus illustrating a significant difference between the two groups.

**Table 1:** Baseline characteristics in the severe OSD cases in which the OSGS was evaluated

	SJS			Chemical and thermal burns			OCP			Toal		
No reoperation:− ,reoperation:+	+	−	*P*	+	−	*P*	+	−	*P*	+	−	*P*
No. of eyes	15	68		4	28		4	60		23	156	
Age(yrs), mean ±SD *	54.9±13.0	52.2±19.0	0.827	43.0±16.3	36.6±15.6	0.549	66.3±8.2	75.3±9.6	0.073	54.8±14.2	58.2±21.1	0.188
Sex, n(%) *			0.151			0.238			1			0.178
Male	3(20.0)	28(41.2)		3(75.0)	27(96.4)		2(50.0)	26(43.3)		8(34.8)	81(51.9)	
Female	12(80.0)	40(58.8)		1(25.0)	1(3.6)		2(50.0)	34(56.7)		15(65.2)	75(48.1)	
*OSGS,mean ±SD*												
OSGS total score	23.5±4.85	18.5±6.03	**0.004**	15.5±1.73	16.07±5.04	0.51	20.5±6.45	17.43±5.42	0.352	21.5±5.52	17.65±5.67	**0.006**
*Cornea*												
Corneal Epithelial Defect	0.67±1.05	0.12±0.44	**0.008**	1.25±1.50	0.50±1.11	0.206	0.25±0.50	0.20±0.61	0.514	0.70±1.06	0.22±0.67	**0.003**
POV	3.0±0.00	2.60±0.88	0.069	3.00±0.00	2.57±0.79	0.244	2.50±0.58	2.68±0.68	0.285	2.91±0.29	2.63±0.79	0.120
Conjunctivalization	2.6±0.74	2.41±0.95	0.578	2.00±1.15	2.21±0.99	0.731	2.25±0.96	2.15±0.86	0.836	2.43±0.84	2.28±0.93	0.435
Corneal Neovascularization	2.33±0.82	2.35±0.96	0.693	1.75±0.96	2.00±1.05	0.589	2.50±1.00	1.93±0.92	0.241	2.26±0.86	2.13±0.98	0.619
Corneal Opacification	2.13±1.12	1.74±0.97	0.114	2.00±1.41	1.79±1.03	0.695	2.00±1.41	1.20±1.02	0.198	2.09±1.64	1.54±1.03	**0.018**
Corneal Keratinization	0.47±0.99	0.41±0.95	0.931	0.00±0.00	0.00±0.00	1	0.25±0.50	0.12±0.56	0.137	0.35±0.83	0.22±0.73	0.332
*Conjunctiva*												
Conjunctival Hyperemia	1.67±0.90	0.79±0.70	**0.001**	2.00±1.15	1.32±0.94	0.271	1.50±0.58	0.63±0.76	**0.022**	1.70±0.88	0.83±0.80	**<0.001**
Symblepharon Formation	1.80±0.86	1.66±0.75	0.600	0.50±0.58	1.32±0.77	**0.049**	2.25±0.50	1.57±0.70	**0.046**	1.65±0.93	1.56±0.74	0.610
Upper fornix shortening	2.20±0.86	1.75±0.85	0.076	0.50±0.58	1.14±0.89	0.173	1.75±1.26	1.78±0.69	0.809	1.83±1.07	1.65±0.83	0.349
lower fornix shortening	2.07±0.70	1.66±0.96	0.113	0.5±1.00	1.25±0.93	0.135	1.50±1.73	1.95±0.79	0.711	1.70±1.11	1.70±0.92	0.839
*Eyelid*												
Trichiasis	2.07±0.80	1.07±0.74	**<0.001**	0.25±0.50	0.82±0.94	0.251	1.50±1.00	1.22±1.04	0.583	1.65±1.03	1.08±0.91	**0.012**
Mucocutaneous Junction Involvement	2.47±0.74	1.93±0.74	**0.013**	1.75±0.50	1.14±0.89	0.110	2.25±0.50	2.00±0.76	0.531	2.30±0.70	1.81±0.83	**0.008**

*OSD* ocular surface disorder, *OSGS* ocular surface grading score, *SJS* Stevens-Johnson syndrome, *OCP* ocular cicatricial pemphigoid, yrs: years, *POV* palisades of Vogt

*Some overlapping

Continuous variables: Wilcoxon rank-sum test

Categorical variables: Fisher's exact test

For each OSGS component, corneal epithelial defect, corneal opacification, conjunctival hyperemia, trichiasis, and mucocutaneous junction involvement were significantly higher in the reoperation group (i.e., *P* = 0.003, *P* = 0.018, *P* < 0.001, *P* = 0.012, and *P* = 0.008, respectively) (Table 1). In the logistic regression analysis, only conjunctival hyperemia had a statistically significant high odds ratio (OR) of 2.65 (95% CI: 1.34-5.22) for reoperation (*P* = 0.005) (Table 2).

**Table 2:** Logistics regression analysis, OSGS in severe OSD cases, and combined surgical methods with AMT

Variables	OR	95%CI					*P* value
*Disease*							
Chemical/thermal burns	1						
SJS	3.35	[0.51, 21.91]					0.081
OCP	1.16	[0.15, 9.19]					0.545
*Cornea*							
Corneal epithelial defect	0.88	[0.38, 2.04]					0.765
POV	1.87	[0.50, 7.01]					0.351
Conjunctivalization	1.02	[0.30, 3.47]					0.970
Corneal neovascularization	0.68	[0.21, 2.24]					0.523
Corneal opacification	1.65	[0.70, 3.94]					0.256
Corneal keratinization	0.83	[0.43, 1.61]					0.587
*Conjunctiva*							
Conjunctival hyperemia	2.65	[1.34, 5.22]					**0.005**
Symblepharon formation	1.23	[0.48, 3.12]					0.664
Upper fornix shortening	0.62	[0.26, 1.47]					0.281
Lower fornix shortening	0.66	[0.34, 1.29]					0.224
*Eyelid*							
Trichiasis	1.72	[0.82, 3.59]					0.153
Mucocutaneous junction involvement	1.60	[0.62, 4.10]					0.328
*Combined surgical methods*							
Other	1						
COMET	0.92	[0.25, 3.36]					0.809
CLET/KEP/LT	0.63	[0.12, 3.22]					0.580

N=179

*OR* odds ratio, *CI* confidence interval

### Correlation among each OSGS component

The correlation coefficient of the changes in the OSGS for each component with a significant difference was examined. Corneal epithelial defect and conjunctival hyperemia were correlated with a correlation coefficient of 0.40. Trichiasis and mucocutaneous junction involvement were correlated with a correlation coefficient of 0.56. In contrast, changes in the OSGS for corneal opacification were not correlated with any other component, and were thus found to be independent of the other components (Table 3).

**Table 3:** Correlation among each OSGS component

Corneal epithelial defect	Corneal opacification	Conjunctival hyperemia	Trichiasis	Mucocutaneous junction involvement	
1.00	0.09	0.40	0.16	0.17	Corneal epithelial defect
	1.00	0.27	0.23	0.27	Corneal opacification
		1.00	0.21	0.12	Conjunctival hyperemia
			1.00	0.56	Trichiasis
				1.00	Mucocutaneous junction involvement

Spearman's rank correlation coefficient

### Combined epithelial transplantation surgery

Epithelial transplantation surgery or LKP performed in conjunction with AMT are shown in Table 4. Of the 156 eyes that did not undergo a reoperation, 63 did not undergo epithelial transplantation (i.e., COMET, CLET, KEP, or LT) and 93 did undergo epithelial transplantation; the mean OSGS of the eyes in each group was 14.9 ± 5.4 and 19.5 ± 5.0, respectively (Table 5). In contrast, of the 23 eyes that underwent reoperation, 9 did not undergo epithelial transplantation (COMET, CLET, KEP, or LT) and14 did undergo epithelial transplantation; the mean OSGS of the eyes in each group was 19.7 ± 4.6 and 22.8 ± 5.5, respectively.

**Table 4:** Rate of reoperation in epithelial transplantation performed in combined with AMT surgery

	COMET	CLET	KEP/LT	LKP
Reoperation−(156 eyes)	57	6	31	7
Reoperation+(23 eyes)	11	0	3	0
Total	68	6	34	7

*AMT* amniotic membrane transplantation, *COMET* cultivated oral mucosal epithelial transplantation, *CLET* cultivated corneal limbal epithelial transplantation, *KEP* keratoepithelioplasty, *LT* limbal epithelial transplantation, *LKP* lamellar keratoplasty

**Table 5:** Total OSGS between the cases with epithelial graft surgery and the cases without epithelial graft surgery and reoperation

	COMET/CLET/KEP/LT−	COMET/CLET/KEP/LT+
reoperation−	63(87.5%) [14.9±5.4]	93(86.9%) [19.5±5.0]
reoperation+	9(12.5%) [19.7±4.6]	14(13.1%) [22.8±5.5]
Total	72 [15.5±5.5]	107 [20.0±5.2]

*The OSGS is indicated in [ ]

## Discussion

In severe OSDs that undergo treatment with AMT, we found that preoperative conjunctival hyperemia (OR = 2.65) is an apparent risk factor for reoperation, and to the best of our knowledge, this is the first report to indicate that preoperative hyperemia is a risk factor of re-AMT in severe OSD cases. Conjunctival hyperemia is one major finding of more severe OSD [1–4, 21, 22], often accompanied by inflammation of the OS. Reportedly, conjunctival hyperemia is correlated to the expression of inflammatory chemical mediators [23, 24]. Hence, the potential exists for excessive inflammation to develop in severe OSD cases with preoperative hyperemia, even after surgery, which would lead to unfavorable conditions, ultimately resulting in reoperation. Using a rabbit model, Tsai reports that uncontrolled postoperative inflammation deteriorated the outcome of limbal transplantation [25]. Moreover, it is reported that in glaucoma surgery (trabeculectomy), inflammation is a risk factor for bleb scarring [26, 27]. From those findings, perioperative inflammation including hyperemia can be considered an important risk factor for reoperation in patients undergoing OS reconstruction.

For successful reconstruction of the OS, it may be important to control preoperative conjunctival hyperemia, and it can also be suggested that in cases with uncontrollable severe hyperemia, reconstructive surgery should not be performed until the OS inflammation is decreased and stabilized. In this study, univariate analysis findings reveal that the OSGS of other preoperative findings, such as corneal epithelial defects, corneal opacification, trichiasis, and mucocutaneous junction involvement, was also significantly higher in the reoperation group. Surgeons should pay close attention to these factors prior to surgery, and specifically, corneal epithelial defects should be addressed, as they seem relatively correlated with conjunctival hyperemia.

In this study, the reoperation rate of AMT (used as a graft) in severe OSD cases was 12.8%, not much different from the 10% to 44.4% widely reported in previous studies. In detail, previous studies report that the reoperation rate of AMT for SJS was 18.1%, for chemical and thermal burns it was 12.5%, and for OCP it was 6.25% [28, 29, 30, 31].

Generally, when AMT is performed, the transplanted AM is denuded (the epithelium is removed). Denuded AM has low immunogenicity, promotes the proliferation of host epithelial cells, and maintains a higher clonogenicity [32, 33]. However, it is reported that denuded AM is not sufficiently effective for severe OSD cases with stem cell exhaustion due to a lack of epithelium. As we previously reported, COMET provides oral mucosal epithelial cells on the AM [16, 17], so there is a possibility that these drawbacks can be improved. For example, Komai et al. report that COMET is safe and effective for symblepharon release and long-term fornix reconstruction in eyes with chronic cicatrization [34].

In this current study, the group with combined epithelial transplantation had a higher preoperative OSGS. Usually, cases in which a combined corneal epithelial transplantation is necessary have progressed to severe OS conditions, and that leads to a high OSGS. Since the reoperation rate was similar for patients with and those without combined epithelial transplantation, our findings suggest that the effect of the epithelial transplantation combination procedure leads to a better postoperative outcome in cases that require OS reconstruction.

The current study did have limitations. First, the number of reoperations does not fully reflect the number of unfavorable conditions post-surgery. For example, since there may have been cases in which the patient was unable to undergo a medical examination during the long-term follow-up course due to old age or a distant condition (especially OCP), it is possible that those subjects were dropped out despite the need for a reoperation. Second, this study was retrospective. It will be necessary to perform prospective studies in the future to obtain a better evaluation.

In conclusion, this retrospective study revealed that preoperative conjunctival hyperemia is a risk factor for reoperation of AMT in severe OSD cases. Moreover, epithelial transplantation may need to be combined with AMT in severely advanced cases of OSD with high OSGS scores.
